# Minimally invasive approaches for the early detection of endometrial cancer

**DOI:** 10.1186/s12943-023-01757-3

**Published:** 2023-03-17

**Authors:** Yufei Shen, Wenqing Yang, Jiachen Liu, Yu Zhang

**Affiliations:** 1 Department of Gynecology, Xiangya Hospital, Central South University, Changsha, Hunan China; 2 Gynaecology Oncology Research and Engineering Central of Hunan Province, Changsha, Hunan China; 3 The Center of Systems Biology and Data Science, School of Basic Medical Science, Central South University, Changsha, Hunan China

**Keywords:** Endometrial cancer, Early diagnosis, Minimally invasive, Biofluid, Biomarkers

## Abstract

Endometrial cancer (EC) is one of the most common gynecologic cancers and its incidence is rising globally. Although advanced EC has a poor prognosis; diagnosing EC at an earlier stage could improve long-term patient outcomes. However, there is no consensus on the early detection strategies for EC and the current diagnostic practices such as transvaginal ultrasound, hysteroscopy and endometrial biopsy are invasive, costly and low in specificity. Thus, accurate and less invasive screening tests that detect EC in women with early stages of the disease are needed. Current research has revolutionized novel EC early detection methodologies in many aspects. This review aims to comprehensively characterizes minimally invasive screening techniques that can be applied to EC in the future, and fully demonstrate their potential in the early detection of EC.

## Introduction

Endometrial cancer(EC) is the most common gynecologic cancer and the fourth most common malignancy among women in developed countries [1]. In 2020, there were 417,367 new diagnoses and 97,370 new deaths in the world [2]. The incidence and associated mortality rates of EC ubiquitously increase worldwide and are projected to rise during the next 10 years [3, 4]. Traditionally, EC is considered to have a good prognosis during the early stage. The 5-year survival rate of patients with stage I EC can be as high as 80%-90%, while the 5-year survival rate is only 50%–65% for stage III and 15%-17% for stage IV [5, 6]. As early diagnosis is associated with a better prognosis, accurate initial diagnosis and timely treatment are key in the management of EC. More importantly, as the follow-up therapy depends mainly on the stage of the disease, early detection of EC can reduce the need for extensive surgical scope or adjuvant treatments, thereby reducing cost, morbidity, and mortality. The target population of early detection for EC is mainly high-risk people (those with obesity, lifetime exposure to unopposed estrogen, metabolic syndrome or Lynch syndrome [7]) and those with symptoms (eg, abnormal postmenopausal bleeding, persistent or recurrent uterine bleeding) suggestive of EC. Unfortunately, there is no EC early detection test accurate and reliable enough to be implemented for triaging high-risk women with suspected EC. The most common method transvaginal ultrasound(TVU) has an extremely high negative predictive value (99%), so it is a reasonable first approach in the early detection of EC but the relatively low specificity means additional tests are needed to rule out endometrial malignancy [7–9]. Endometrial biopsy is cost-effective but discomfort and false-negative results are the most common complications [10, 11]. Endometrial biopsy under hysteroscopy is less invasive but pain and vasovagal episodes can contribute to the failures in hysteroscopy [12]. Therefore, there is an urgent need for accurate and less invasive approaches to use in the early detection of EC.

**Fig. 1:**
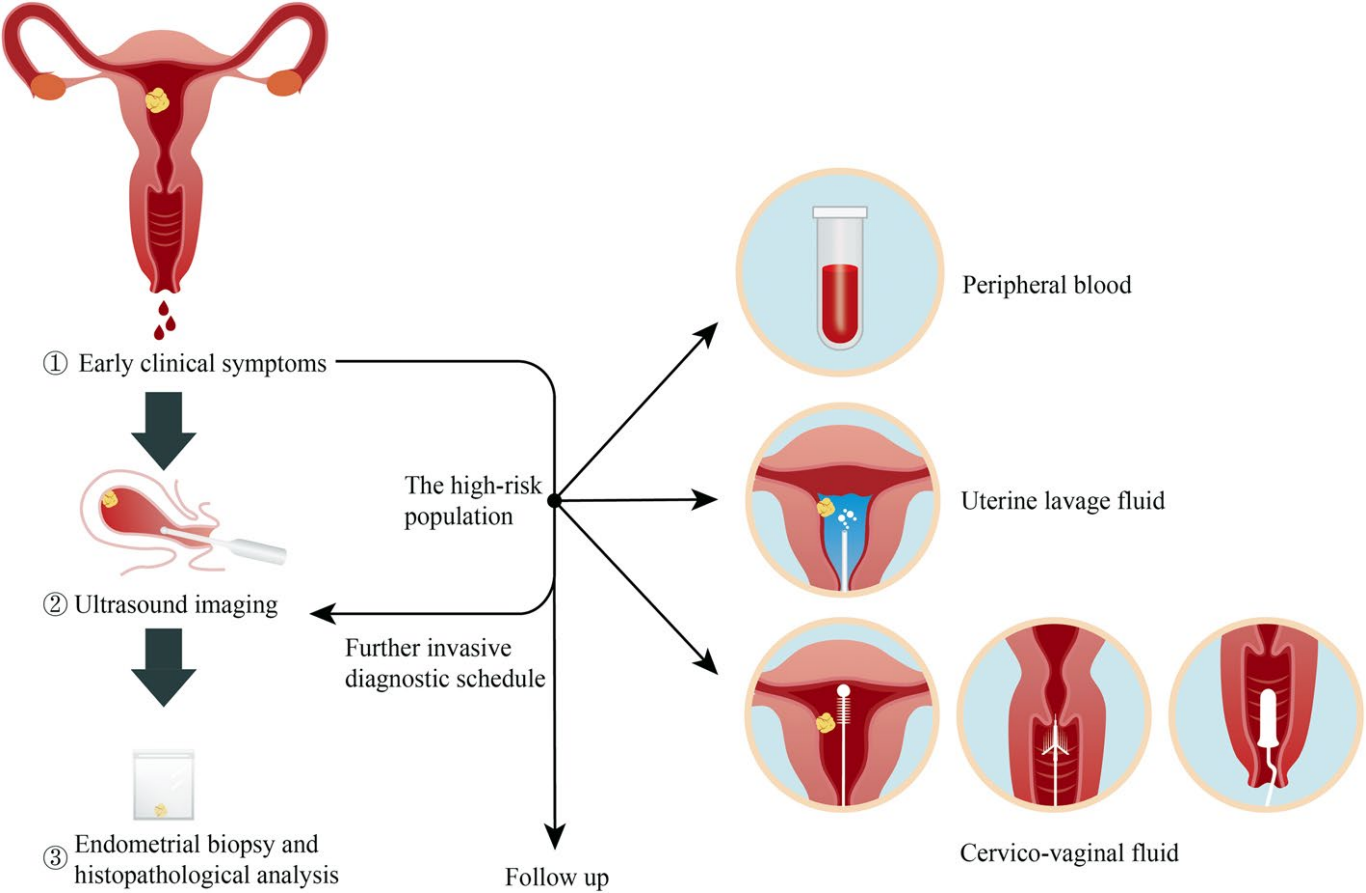
Minimally invasive approaches in the diagnostic schedule of EC

Peripheral blood, uterine lavage, cervicovaginal fluid and other potential tumor-specific biofluids can be collected minimally invasively [7, 8]. Gene sequencing in the biofluid can detect tumorigenic DNA, which may lead to early detection of tumors and thus achieve early diagnosis of EC [13]. A minimally invasive method combing with gene sequencing technologies to solve the problem of early detection of EC is a hot spot of current research and the direction of future development. Research in the past decades has revolutionized this field in many aspects. We hope not only can minimally invasive approaches potentially benefit current diagnostic schedule among high-risk women and those with symptoms, but they may also be potentially appropriate in screening of asymptomatic populations and furthermore in the general population in the future. In this review, we will first summarize the current methods of EC diagnosis, with their advantages and limitations. Then the progress in minimally invasive approaches that can be applied to EC early detection in the future will be reviewed, pointing out the shortcomings of existing research and highlighting the future direction and focus (Fig. 1).

### Current methods of EC diagnosis

#### *Early clinical symptoms*

An important clinical feature of EC or precancerous lesions is abnormal postmenopausal bleeding(PMB), which occurs more than one year after menopause and manifests as dripping bleeding, bloody leucorrhea, and contact bleeding [14]. However, although early detection strategies focused on women with PMB have the potential to screen as many as 90% of endometrial malignant diseases, only 5–10% of women with PMB will be diagnosed with malignant pathology [15]. Perimenopausal and premenopausal EC patients may present with menstrual cycle disorders and abnormal uterine bleeding (AUB) [16]. Still, 90% of the patients have PMB for non-cancer etiology and about 50% of the women have PMB secondary to polyp, which can be easily diagnosed and treated under endometrial biopsy or hysteroscopy [17]. As a result, endometrial evaluation (including ultrasound imaging, endometrial biopsy with or without hysteroscopy, together with the histopathological examination) for subtle pathology is proposed in all patients with above abnormal symptoms, so the costs for diagnostic evaluation of AUB and PMB are substantial [18].

### *Ultrasound imaging*

Ultrasound imaging especially transvaginal ultrasonography (TVU) is a safe and well-tolerated method for potential EC patients. It can detect endometrial abnormalities including endometrial thickening and abnormal imaging features(cystic endometrium, fluid in the cavity, suspected polyps, or other suspicious features), which are associated with an increased risk of EC [19]. Several multicenter trials have confirmed that TVU is enough for an initial evaluation of PMB if the TVU reveals a ≤ 4 mm endometrial echo, given that the rate for endometrial cancer drops below 1% when the endometrial thickness (ET) is less than 4 mm [9, 20]. However, the test specificity is relatively poor, which inevitably causes an increase in subsequent invasive examinations and biopsies, aggregating psychological and financial burdens [9]. What’s more, as TVU needs trained specialists to perform, the results may be subjective and unstable. For example, an axial uterus, adenomyosis, coexisting myomas, or previous surgery history can lead to unreliable results assessed by TVU [20–22]. In premenopausal and perimenopausal women, simple measurement of ET has some limitations due to physiological sex hormone changes [23]. There is still no consensus on the ideal ET cut-off for these patients [24], so alternative, non-invasive triage tools are needed to help physicians to decide on further examinations. Saline infusion Sonohysterography (SIS) is also a safe procedure for endometrial evaluation [25]. It can provide a clearer picture of the uterine cavity and improve the diagnostic accuracy of endometrial lesions than TVU, especially focal endometrial abnormalities [26]. So it is usually an alternative when TVU fails to identify a thin, distinct endometrial echo [27]. However, due to technical and cost requirements, SIS is less commonly used in clinical practice [28].

It is worth noting that type I EC (associated with unopposed estrogen stimulation and has a favorable prognosis) is expected to be associated with a thickened endometrium and endometrial hyperplasia, but type II EC (not estrogen-driven and has an unfavorable prognosis) often arise independently of hyperplasia [29, 30]. Studies have found that 25%-34% of type II EC patients have a thin or indistinct endometrial echo, which means the use of TVU is limited in type II EC [31, 32]. So further evaluation of the endometrium regardless of endometrial thickness is needed when persistent or recurrent uterine bleeding occurs.

### *Endometrial biopsy and histopathological analysis*

Collecting sufficient endometrial tissue to conduct a histological analysis is one of the standard methods for EC diagnosis. There are various ways to get endometrial tissue histology: pipelle endometrial sampling, dilation and curettage(D&C), endometrial biopsy under hysteroscopy and others. The pipelle, first described in 1984 by Cornier [33], can obtain endometrial tissue through negative pressure and the accuracy can exceed 95% [34, 35]. The Pipelle endometrial sampler is low in cost, causes minimum discomfort and carries few side effects [36–38]. The major disadvantage is a higher sampling failure rate than D&C caused by an inability to access the uterine cavity or insufficient amount of tissue collected [39]. What’s more, various subsequent studies point out that Pipelle has a limited ability to identify focal lesions and is only suitable for homogeneous endometrium [40, 41]. D&C is one of the standard methods for the evaluation of suspected endometrial lesions. It is equally effective but more costly than pipelle endometrial sampling. The anesthetics side effects, infection and perforation caused by the D&C procedure also limit its use [37]. In recent years, hysteroscopy, a less invasive and accurate endoscopic technique providing a satisfactory assessment of the uterine cavity has merged as a powerful tool in endometrial biopsy [12]. It allows for direct visual localization of suspicious lesions for biopsy or excision, which is highly accurate in the diagnosis of EC and can reduce the false negative rate [42, 43]. Despite many advantages, complications associated with the procedures are inevitable. Cervical stenosis and pain are the main reasons for incomplete and failed hysteroscopy [44]. Vasovagal reaction, local anesthetic toxicity, uterine perforation, fluid overload and uterine hemorrhage may occur, but these remain incredibly rare events and preoperative use of misoprostol, stabilize the power of loop, careful monitoring of the collected irrigating medium can help prevent these complications [45]. SIS-guided endometrial aspiration is not a first-line method in endometrial biopsy because it doesn’t improve the diagnostic performance along with infection and tumor dissemination risk [46, 47]. It is only suitable for patients whose diagnosis is not clear after biopsy or for those with D&C and hysteroscopy contraindications [48, 49]. After sufficient tissue is obtained, the endometrial histopathological analysis will be done to determine the pathological diagnosis of patients, providing information about judgment of benign or malignant lesions to choose optimal treatments. Further molecular tests can help with the accurate diagnosis of premalignant and malignant lesions [50, 51].

### Novel minimally invasive approaches in the early detection of EC

The current diagnostic flow for EC involves sequential, invasive tests to assess the ET by TVU, visualize the endometrial cavity under hysteroscopy, and finally do an endometrial biopsy for histopathological analysis [52]. However, methods discussed above indiscriminately focus on women presenting with symptoms because they are more likely to have underlying EC or precursor lesions [22]. So far, gynecologists haven’t reached a consensus on the early detection program of EC in either the general population or specific high-risk groups. An ideal early detection flow should be accurate, cost-effective and patient-friendly. The main goal is to identify high-risk patients for invasive diagnostic schedules while safely reassuring low-risk women. More acceptable screening tools, including blood, uterine lavage, and cervicovaginal fluid show promise, and novel genomic biomarkers detected in biofluid samples may be key to early screening [7]. Innovations in EC diagnostics include the usage of minimally invasive specimen collection techniques and the identification and validation of cancer-specific biomarkers that can be detected in non-invasive biofluids, which have now become one of the main focuses of research [53]. It has numerous advantages over traditional biopsy, including convenience, minimal invasiveness, real-time, and reproducibility, showing its powerful superiority in the management of patients who have difficulty obtaining biopsy tissue. In addition to blood samples, some other body fluids such as saliva, urine, cerebrospinal fluid, and even feces can also be used as sources [54, 55] (Table 1).

**Table 1:** Minimally invasive specimen collection techniques in this review

Author	Biospecimen	Number of EC cases	Number of controls	Technology	Findings
Tanaka2012 [56]	Blood	53	9	qPCR	The cfDNA levels in ECs tended to be higher than in benign condition
Vizza2018 [57]	Blood	60	22	qPCR-Alu115 to measure cfDNA content and qPCR-Alu247 to measure DNA integrity index	Total cfDNA content significantly increases in high grade EC. Serum DNA integrity was higher in samples with LVSI
Łukasiewicz2021 [58]	Blood	53	242	QIAseq Targeted Human Colorectal Cancer Panel covering 71 genes	Platelet-dedicated classifier yielded AUC of 97.5% in the test set when discriminating between healthy subjects and cancer patients. ctDNA-dedicated classifier discriminated primary tumor tissue samples with AUC of 96%
Torres2013 [59]	Blood	77	45	Agilent Human miRNA Microarray to investigate the expression of miRNAs	miR-9/miR-1228 and miR-9/miR-92a, classified EEC plasma samples with high accuracy yielding AUCs of 0.90and 0.913, respectively
Jia2013 [60]	Blood	33	42	TaqMan® low-density arrays and hydrolysis probe-based stem-loop qRT-PCR	The qRT-PCR analysis identified a profile of four serum miRNAs (miR-222, miR-223, miR-186 and miR-204) as a fingerprint for EEC detection(ROC = 0.927)
Maritschnegg2015 [61]	Uterine lavage	5	27	Multiplex amplification and sequencing of 136 amplicons covering 16 genes	All five analyzed lavage specimens from patients with EC harbored mutations. Eight (29.6%) of 27 patients with benign lesions tested positive for mutation
Nair2016 [62]	Uterine lavage	7	95	Ultra-deep targeted next-generation sequencing gene panels composed of 56 + 12 genes	EC driver mutations were identified in all seven women who received a cancer diagnosis
Fiegl2004 [63]	Tampons	15	109	DNA methylation changes (MethyLight) of 5 genes	All endometrial cancer patients revealed three or more methylated genes, whereas 91% (99 of 109) of the patients without EC had no or fewer than three genes methylated in their vaginal secretion
Bakkum-Gamez2015 [64]	Tampons	38	28	Pyrosequencing for DNA methylation of 12 genes	Mean methylation was higher in tampon specimens from EC for 9 of 12 genes; AUC was highest for HTR1B (0.82), RASSF1 (0.75), and HOXA9 (0.74)
Jones2013 [65]	vaginal swabs	64	23	Epigenome-wide methylation analysis of > 27,000 CpG sites	HAND2 is one of the most commonly hypermethylated and silenced genes in endometrial cancer
Doufekas2016 [66]	vaginal swabs	30	73	Illumina 450 k DNA methylation bead array assay	The EC DNAme signature resulted in ROC area under the curve of 0.83 to discriminate controls and the cancers
Kinde2013 [67]	Pap smear	24	14	The modified Safe-SeqS assay used to detect mutations in 12 different genes	Scientists were able to identify the same mutations in the DNA from liquid Pap smear specimens in 100% of EC (24 of 24)
Wang2018 [68]	Pap smear	382	714	The modified Safe-SeqS assay to detect mutations in 18 genes, and an assay for aneuploidy	81% of 382 EC patients were positive, including 78% of patients with early-stage disease
Wang2018 [68]	Tao brush	123	125	The modified Safe-SeqS assay to detect mutations in 18 genes, and an assay for aneuploidy	93% of 123 patients with EC were positive, whereas none of the samples from 125 women without cancer were positive (specificity, 100%)
Huang2017 [69]	Cervical scrapings	50	96	quantitative methylation-specific PCR (QMSP) for 14 genes	A panel comprising any two of the three hypermethylated genes(BHLHE22, CDO1, CELF4) reached a sensitivity of 91.8%, specificity of 95.5% for EC screening
Chang2018 [70]	Cervical scrapings	40	40	Quantitative methylation-specific polymerase chain reaction for 14 genes	Sensitivity and specificity of POU4F3/MAGI2 were 83%-90% and 69%-75% for detection of EC

### *Peripheral blood*

The blood sample is a well-accepted source to collect for early cancer diagnosis. However, to date, no suitable serum biomarker for early diagnosis of EC is available because of low concentration and dissatisfied accuracy. Only HE4 may have some reference value in the diagnosis of EC [71]. So researchers have turned their attention to the gene information of circulating tumor components in the blood including circulating tumor cells, plasma cell-free DNA (cfDNA), circulating tumor DNA (ctDNA), and circulating miRNAs [72, 73]. Some studies have evaluated the density of cfDNA in benign gynecologic diseases and EC patients and found that the cfDNA levels in ECs tend to be higher than that in benign conditions [56]. Furthermore, the increase in cfDNA levels is more pronounced in high-grade EC [57]. The DNA released by dying cells into the blood is called plasma cfDNA and ctDNA is the tumor-derived fraction of cfDNA [74]. The ctDNA test serves a promising role in the early detection of EC. A 4-gene panel(CTNNB1, KRAS, PTEN, and PIK3CA) has been used to test ctDNA in 48 patients with EC and detected somatic mutations consistent with tumor tissue in 33% of the patients [75]. Using RNA-sequencing and DNA-sequencing, both tumor-educated platelets (TEPs) and ctDNA can discriminate between healthy controls, benign gynecologic conditions, and EC according to Marta’s study [58]. The latest research in 2022 found that hypermethylated ctDNA ZSCAN12 and/or oxytocin allow the detection of patients with EC with high diagnostic specificity/sensitivity(> 97%; AUC = 0.99) [76]. miRNA is a class of endogenous short non-coding RNA molecules widely expressed in cells and can involve numerous processes such as tumor cell proliferation, differentiation, and apoptosis [77]. Circulating miRNA exists in the form of nucleic acid-protein complexes or is encapsulated in the form of exosomes. Circulating miRNAs and exosome miRNAs have been confirmed useful in the diagnostics of different types of cancer and can become biomarkers for diseases [78–80]. Various biomarkers such as miR-99a/miR-199b [81], miR-9/miR-1228/miR-92a [59], hsa-miR-200c-3p [82] and miR-222/miR-223/miR-186/miR-204 [60] have been discovered to hold a great promise to become noninvasive biomarkers for early EC detection. However, not all genetic mutations in tumor can be detected in blood, especially in the early stage of tumor. In terms of early EC diagnosis, the technology to diagnose EC by gene sequencing of circulating tumor components in the blood alone has yet to mature.

### *Uterine lavage fluid*

Uterine lavage, which can direct contact with tumor is an ideal source of biofluids. In 2015, Maritschnegg’s group first proposed that lavage of the uterine cavity could detect shedding EC cells. The DNA obtained from each lavage sample was examined for the presence of somatic mutations using massively parallel sequencing and lavage samples were classified as positive when one or more mutations were detected. Finally, all uterine lavage specimens from patients with stage IA EC harbored mutations despite a small sample size of five patients [61]. A prospective study in 2016 used targeted gene sequencing to detect somatic mutations in uterine lavage fluid obtained from women undergoing hysteroscopy [62]. Endometrial driver mutations (three PTEN mutations, one PIK3CA mutation, one CTNNB1 mutation, and one FBXW7 mutation) were identified in all seven women who received a cancer diagnosis after gold-standard histopathology, suggesting that NGS-based analysis of uterine lavage can achieve satisfied sensitivity for EC diagnosis. However, uterine lavage can cause significant discomfort to patients because it needs to be collected during hysteroscopy [22]. Professional equipment and trained personnel are required to perform uterine lavage safely, weakening its advantages over current diagnostics and restricting its clinical applications.

### *Cervicovaginal fluid*

The cervicovaginal fluid contains natural tumor cells shedding into lower genital tract and can be collected minimally invasively. The presence of cancer-associated mutations or the methylation levels of DNA in the cervicovaginal fluid can help to detect the EC. Pap smears are routinely used for cervical cancer screening in the general population due to low discomfort and high acceptance, leading scientists to become interested in its diagnostic value in EC. However, it was later confirmed that the Pap smear is not an effective screening tool for EC because the amount of shedding tumor cells is usually very slight in the area sampled for the Pap smear [83, 84]. In 2013, Kinde innovatively used the traditional Pap smear to collect DNA for the detection of somatic mutations present in EC tumor cells accumulated in the cervix [67]. This finding subsequently led to the development of the “PapGene” test, a sequenced-based 12-gene-panel(APC, AKT1, BRAF, CTNNB1, EGFR, FBXW7, KRAS, NRAS, PIK3CA, PPP2R1A, PTEN, and TP53) for the routine medical screen of EC. In 2018, the group updated the “PapGene” test to the “PapSEEK” test, incorporating assays for mutations of 18 genes(AKT1, APC, BRAF, CDKN2A, CTNNB1, EGFR, FBXW7, FGFR2, KRAS, MAPK1, NRAS, PIK3CA, PIK3R1, POLE, PPP2R1A, PTEN, RNF43, and TP53) and aneuploidy [68]. The PapSEEK can successfully identify 81% of EC patients(78% early-stage) with a pap brush, and the specificity exceeds 99%. Tao brush, inserted into the uterine cavity at the level of the fundus is an improvement of pap brush despite certain drawbacks because of its relatively high cost and high unsuccessful insertion rate [38]. The use of a Tao brush [85] can further increase the sensitivity to 93% and specificity to 100%, which is verified in subsequent studies [86, 87].

Various proof-of-concepts studies have confirmed that the epigenomic analyses of DNA collected from tampons, vaginal swabs or cervical scrapings can help to diagnose EC. The intravaginal tampon is a noninvasive and well-accepted absorbent hygiene product for women. Bakkum-Gamez et al. [64], collected vaginal pool samples with tampons and found nine genes hypermethylated in EC patients. According to Fiegl’s study the methylation status of DNA obtained from tampons could successfully identify EC out of other unmalignant diseases(sensitivity 100%, specificity 97.2%) [63]. This self-collected method can enable women to collect and deliver the specimen to a laboratory easily, overcoming socioeconomic status and geographical barriers. In addition, serial collection of tampons can enable us to monitor high-risk patients in a long term. Besides tampons, cervical scrapings and vaginal swabs are also good sources of DNA for molecular testing. They can be obtained easily during each outpatient visit, are low in cost and are virtually non-invasive [88]. A certain amount of research proved that DNA methylation of tumor driver genes detected in cervical scrapings and vaginal swabs have considerable sensitivity and specificity in the diagnosis of EC [65, 66, 69, 70, 89]. The latest study in 2022 described the WID-qEC test, a three-marker test that evaluates DNA methylation in gene regions of GYPC and ZSCAN12 [90]. In cervical smear, self-collected, and vaginal swab samples derived from symptomatic patients, it could detect EC with sensitivities of 97.2% (95% CI, 90.2 to 99.7), 90.1% (83.6 to 94.6), and 100% (63.1 to 100), respectively. However, the existing studies are based on previously diagnosed women, so the influence of examinations during the diagnostic process cannot be ignored. A series of diagnostic procedures before sampling may increase the shedding of tumor-related cells into the lower genital tract, exaggerating its actual effect in the real world. More large prospective cohort studies of undiagnosed women are needed.

### Challenges and future directions

There is no denying that the invention of minimally invasive biofluid sample collection techniques has broadened the horizons of early cancer detection, with many encouraging examples in other tumors [91, 92]. There are many active avenues of research in the field of early detection for EC, and exciting advancements are being made. However, the clinically available early detection flow for EC is still on the way. First, the studies mentioned above are primarily pilot or retrospective studies with a limited number of cases. Further prospective discovery work and validation studies are needed to further validate the value of those methodologies. Second, the concordance between tumor tissue and biofluids are not consistent across different studies because of different disease stage, tumor type, and tumor heterogeneity [55, 93]. What’s more, published studies often use different criteria to select gene panels for early detection of EC, and it is challenging to find the optimal panel to be applied in the target population. Third, the detection of precancerous lesions is important because a third of women with atypical hyperplasia can find concurrent EC after surgery and the diagnosis of precancerous lesions remains challenging and subjective in some cases [94, 95]. However, the early detection of precancerous lesions will possibly cause unnecessary intervention and definitely long term anxiety. The application of minimally invasive biofluid sample collection techniques and further improvement of the sensitivity and specificity of diagnostic tests may solve these issues. A balance needs to be found in the pros and cons of early detection of precancerous lesions. But Last but not least, metabolomics which assesses the qualitative and quantitative of serum metabolomes in patients has been emerging as a novel invasive way for early screening of EC, so the application potential of metabolomics deserves more research [96, 97]. Among the above minimally invasive approaches, cervicovaginal fluid shows the greatest potential for clinical application of EC early detection in the future. It is virtually non-invasive, without outpatient operation and more cost-effective. Compared to peripheral blood and uterine lavage fluid, higher concentrations and less dynamic changes of tumor-associated biomarkers can be found in the cervicovaginal fluid, resulting in higher sensitivity and specificity [98]. Although sequencing of somatic mutations(often limited to subclones of tumor cells) in the cervicovaginal fluid is effective in the early detection of EC, DNA methylation test(widespread across the tumor tissue, overcoming lower sensitivity due to tumor heterogeneity) shows greater strengths and potential because epigenetic alteration happens earlier in tumorigenesis, and is more stable [99]. In the nearby future, the minimally invasive biofluid sample collection technique can not only benefit high-risk women and those presenting with abnormal clinical symptoms suggestive of EC for further triage for malignancies, it may also help with the follow-up of fertility-sparing young patients with grade 1 EC limited to the endometrium or atypical endometrial hyperplasia [100]. Those minimally invasive approaches can help to assess the effectiveness of fertility-sparing treatment with fewer invasive intrauterine operations, which is beneficial to patient's future pregnancy.

To conclude, this review demonstrates the great potential of minimally invasive approaches in the early detection of EC. The application of the above techniques in the management of EC yields a broad research value. The current research evidence is limited and further clinical validation in large clinical trials is needed.

## Data Availability

Not applicable.
