# Correction: minimally invasive approaches for the early detection of endometrial cancer

**DOI:** 10.1186/s12943-023-01777-z

**Published:** 2023-04-26

**Authors:** Yufei Shen, Wenqing Yang, Jiachen Liu, Yu Zhang

**Affiliations:** 1 Department of Gynecology, Xiangya Hospital, Central South University, Changsha, Hunan China; 2 Gynaecology Oncology Research and Engineering Central of Hunan Province, Changsha, Hunan China; 3 The Center of Systems Biology and Data Science, School of Basic Medical Science, Central South University, Changsha, Hunan China

**Correction: Mol Cancer 22, 53 (2023).**

10.1186/s12943-023-01757-3

Following publication of the original article [1], the authors reported that the third authors name should be “Liu Jiachen” instead of “Liu Jiacheng”.

This has been updated above and the original article has been corrected.
